# Cost effectiveness of chemohormonal therapy in patients with metastatic hormone-sensitive and non-metastatic high-risk prostate cancer

**DOI:** 10.1590/S1679-45082017GS4017

**Published:** 2017

**Authors:** Pedro Nazareth Aguiar, Carmélia Maria Noia Barreto, Bárbara de Souza Gutierres, Hakaru Tadokoro, Gilberto de Lima Lopes

**Affiliations:** 1 Faculdade de Medicina do ABC, Santo André, SP, Brazil.; 2 Beneficência Portuguesa de São Paulo, São Paulo, SP, Brazil.; 3 Universidade Paulista, São Paulo, SP, Brazil.; 4 Universidade Federal de São Paulo, São Paulo, SP, Brazil.; 5 Sylvester Comprehensive Cancer Center, Miami University, USA.

**Keywords:** Economics, pharmaceutical, Prostatic neoplasms/drug therapy, Cost control, Public health, Farmacoeconomia, Neoplasias da próstata/tratamento farmacológico, Controle de custos, Saúde pública

## Abstract

**Objective:**

To assess the cost-effectiveness of chemohormonal therapy in patients with metastatic hormone-sensitive and non-metastatic high-risk prostate cancer.

**Methods:**

An analytical decision model was developed to determine the cost-effectiveness of chemohormonal therapy *versus* androgen deprivation therapy alone in patients with metastatic hormone-sensitive prostate cancer and patients with non-metastatic high-risk prostate cancer. The cost-effectiveness in metastatic patients with a high-volume disease was assessed separately. The model used data from randomized clinical trials and drug acquisition costs in Brazil. In addition, the costs of post-progression therapies have been included in this model. The benefits to health are expressed as the quality-adjusted life-years, and the incremental cost-effectiveness ratios were calculated.

**Results:**

Chemohormonal therapy may be associated with improved quality-adjusted life-years for all patient. The improvement was more than six times greater for patients with high-volume metastatic disease. In these patients, the incremental cost-effectiveness ratios were up to 74% lower than the incremental cost-effectiveness ratios of patients with non-metastatic disease.

**Conclusion:**

Chemohormonal therapy has been more cost-effective in patients with high-volume metastatic disease.

## RESUMO

**Objetivo:**

Avaliar a relação custo-efetividade da adição de quimioterapia hormonal em pacientes com câncer de próstata metastático sensível a hormônio ou localizado de alto risco.

**Métodos:**

Um modelo de decisão analítico foi desenvolvido para determinar o custo-efetividade da adição de quimioterapia *versus* a monoterapia de privação de andrógeno para pacientes com câncer de próstata metastático hormônio-sensível e pacientes de alto risco com câncer de próstata não metastático. O custo-efetividade em pacientes metastáticos com um alto volume da doença foi verificado isoladamente. Os dados do modelo foram obtidos de ensaios clínicos randomizados utilizando custos de aquisição de medicamentos no Brasil. Os custos de terapias pós-progressão também foram incluídos no modelo. Os efeitos foram expressos em anos de vida ajustados por qualidade, e foram calculadas as razões de custo-efetividade incremental.

**Resultados:**

A adição de quimioterapia levou a um ganho de anos de vida ajustados por qualidade para todos os doentes. Este incremento foi seis vezes maior para os pacientes com doença metastática de alto volume. Nestes pacientes, as taxas do custo incremental por anos de vida ajustados por qualidade foram até 74% mais baixos do que o aumento das taxas dos pacientes com doença não metastática.

**Conclusão:**

A adição de quimioterapia foi mais custo-efetiva para pacientes com doença metastática de alto volume.

## INTRODUCTION

Prostate cancer is the second most common neoplasm in men worldwide with an estimated 1,100,000 new cases and 307,000 deaths reported in 2012.^(^ ^1^ ^)^ In Brazil, between 2016 and 2017, the *Instituto Nacional de Cancer José Alencar Gomes da Silva* (INCA) estimates that prostate cancer will be the most common neoplasm among men, excluding non-melanoma skin cancers.^(^ ^2^ ^)^

U.S. data indicate that of all prostate cancer cases, 80% are confined to the prostate gland, 12% are locally advanced and invaded regional lymph nodes, and 4% are distant metastases; approximately 4% of the cases have an unknown stage.^(^ ^3^ ^)^ Although the lack of data in Brazil, it is hypothesized that there will be a greater proportion of metastatic disease reported at diagnosis due to socioeconomic reasons.^(^ ^4^ ^)^

The main treatment for metastatic disease has been androgen deprivation therapy (ADT) since mid-1966, when Charles B. Huggins was awarded with the Nobel Prize. He showed that androgen deprivation was an effective treatment in patients with locally advanced or metastatic prostate cancer, with a 15% decrease in the cancer-specific mortality rate.^(^ ^5^ ^)^ However, there was no change in the overall survival (OS) of patients with localized disease.^(^ ^5^ ^)^

Recent studies have reviewed this treatment paradigm and have compared ADT alone *versus* ADT in combination with chemotherapy for patients with localized, advanced, or metastatic disease. The GETUG-AFU 15 study (*Androgen-deprivation therapy alone or with docetaxel in non-castrate metastatic prostate cancer*) did not show any improvement in OS following treatment with ADT plus docetaxel *versus* ADT alone; however, an objective response rate of 28% was achieved in patients who were treated with a combination of ADT and docetaxel.^(^ ^6^ ^)^ The median progression free survival (PFS) for the ADT plus docetaxel group increased to 23.4 months from 18.5 months for the ADT group (*hazard ratio* - HR: 0.75, 95% of confidence interval - 95%CI: 0.59-0.94; p=0.015).^(^ ^6^ ^)^

In addition, the Systemic Therapy in Advancing or Metastatic Prostate Cancer: Evaluation of Drug Efficacy (STAMPEDE) and GETUG-AFU 12 studies have assessed chemotherapy plus ADT for non-metastatic patients with a high-risk of localized disease (*e.g*. elevated prostate specific antigen − PSA at diagnosis and high-grade tumors). In the STAMPEDE study, 24% of the patients did not present with metastatic disease and the results of the study pointed to a benefit in terms of OS and PFS in favor of the combined treatment.^(^ ^7^ ^)^ In the GETUG-AFU 12 study, which only included patients with non-metastatic disease, there was an increase in the recurrence free survival (RFS) in patients that received the combination treatment of chemotherapy plus ADT compared to patients that were treated with ADT alone (HR: 0.71; p=0.017).^(^ ^8^ ^)^

Pharmacoeconomics evaluates the costs and benefits of drug therapy under the following aspects: the cost of this treatment to the health system, how much it improves disease prognoses, the demand and supply of the treatment for a given disease, and the budget.^(^ ^9^ ^)^ The main objective of pharmacoeconomics is to equate the increasing financial demand of new treatments with sustainability, so that the treatment for a specific subset of the population is available for everyone who will benefit the most.^(^ ^9^ ^)^ There are two important concepts to consider: the quality-adjusted life-year (QALY) and incremental cost-effectiveness ratio (ICER).^(^ ^10^ ^)^

Quality-adjusted life-year measures both the number of years gained by taking a treatment and the quality of life during the treatment period. This is measured in terms of the patient’s ability to carry out daily activities. This is the life-years provided by the treatment adjusted to the quality of life score (also known as utility, on a scale of zero, dead, to 1, full capacity).^(^ ^10^ ^)^

Incremental cost-effectiveness ratio evaluates the cost-effectiveness of the treatment intervention, *i.e*., the cost for each QALY gained by the treatment. The value is the ratio between the difference in treatment costs and the QALY gained.^(^ ^10^ ^)^

## OBJECTIVE

To evaluate the cost-effectiveness of adding chemotherapy to androgen deprivation therapy in three distinct subgroups of patients with prostate cancer: patients with metastatic disease, patients with extensive metastatic disease and patients with high-risk non-metastatic disease.

## METHODS

We developed an analytical model to determine the cost-effectiveness of adding chemotherapy to ADT *versus* ADT alone for the initial treatment of prostate cancer. In our model, we compared early chemotherapy plus ADT or ADT alone. The model was developed in the Microsoft Excel Professional Plus 2013.

**Figure 1:**
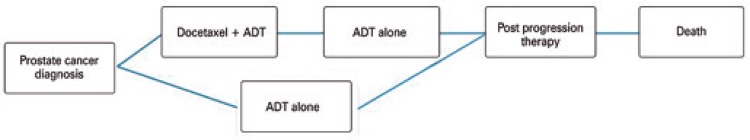
Type of treatment after progression and death

The type of treatment after progression and death (Figure 1) were also included. The same model was applied to patients with hormone-sensitive metastatic disease and high-risk non-metastatic disease. Subsequently, analyses were performed considering only the metastatic patients with a high-volume of disease according to the definitions of the CHAARTED study (ChemoHormonal therapy *versus* androgen ablation randomized trial for extensive disease in prostate câncer): presence of visceral metastasis and/or four or more lesions in the bone with at least one lesion affecting a bone outside of the vertebrae or pelvis.^(^ ^11^ ^)^

ADT: androgen deprivation therapy.

To calculate the QALY of each treatment, the different health states present in the model received a utility score based on the literature.^(^ ^12^ ^)^ The utility score for chemotherapy was obtained from quality of life analyzes previously published.^(^ ^8^ ^,^ ^11^ ^)^ Utility values were reduced according to the adverse events caused by each first-line treatment using the disutility scores available in the literature.^(^ ^13^ ^-^ ^15^ ^)^

The costs for each treatment and the costs of the post-progression therapies were considered. These costs were based on the Brazilian discount price index accessed in June 2016.^(^ ^16^ ^)^ All costs were converted to US dollars based on an exchange rate of R$ 3,25 to US$ 1.00. The costs of adverse events were not considered in the Brazilian model because there has not been enough data published in the local literature to extrapolate the costs from other countries (such as the United States or the United Kingdom), which may not accurately represent the reality in Brazil. All costs included in the analysis are summarized in the table 1.

**Table 1:** Costs summary

Costs	Docetaxel + ADT ($)	ADT alone ($)
Docetaxel	4,733.90	0
ADT	5,441.74	3,886.95
Post progression	9,618.64	9,863.14
Adverse events	NA	NA
Supportive care	NA	NA
Total	19,794.28	13,750.09

NA: not assessed; ADT: androgen deprivation therapy.

Data regarding PFS, or RFS in the case of non-metastatic patients, and OS were extracted from randomized clinical trials.^(^ ^8^ ^,^ ^11^ ^)^ A lifetime mortality estimate was not made since the follow-up time for each study was adequate to demonstrate the difference in outcomes between each type of treatment (8.8 years in the GETUG-AFU 12 study and 28.9 months in the CHAARTED study).^(^ ^8^ ^,^ ^11^ ^)^ The effects were expressed in QALY and ICER.

After analyzing the clinical scenario of each group of patients a probabilistic sensitivity analysis was performed, taking into account the CI of RFS, PFS and OS. In addition, scenarios with 100% increase or 50% discount on the cost of docetaxel cost and QALY gain were considered. These analyses were performed to confirm the robustness of the data and facilitate the comparison of the results between the different patient subgroups. The probabilistic sensitivity analysis results are presented in a Tornado diagram.

## RESULTS

### High-risk non-metastatic disease

**Figure 2:**
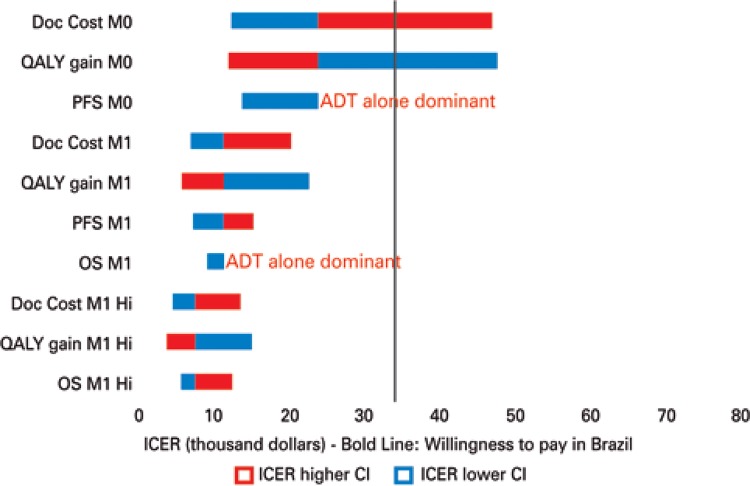
: Tornado diagram for docetaxel plus androgen deprivation therapy *versus* androgen deprivation therapy alone

In the analysis of patients with high-risk non-metastatic disease, the addition of docetaxel to ADT promoted a gain of 0.12 QALY. As a result, the incremental cost of this therapy was US$ 25,929.62 per QALY. In the probabilistic sensitivity analysis, 53% of the scenarios evaluated were cost-effective based on the three-fold gross domestic product (GDP) per capita (US$ 33,000.00) per QALY. In 33% of the scenarios evaluated ADT alone was cost-effective (Figure 2).

Doc: docetaxel; M0: non-metastatic; M1: all metastatic; QALY quality-adjusted life-year; PFS: progression-free survival; OS: overall survival; Hi: metastatic with high volume of disease; ADT: androgen deprivation therapy; ICER: incremental cost-effectiveness ratio; CI: confidence interval.

### Metastatic disease

In the metastatic disease analysis, there was an increase of 0.53 QALY with the addition of docetaxel to ADT. The incremental cost per patient was R$ 11.228.55 per QALY. Almost all of the scenarios evaluated in the probabilistic sensitivity analysis were considered cost-effective (80% of the cases). In the remaining (20%) scenarios, ADT alone was cost effective (Figure 2).

### High-volume metastatic disease

Considering only patients with a high-volume of metastatic disease, there was an increase of 0.70 QALY with the addition of docetaxel to ADT. The incremental cost in this subpopulation was US$ 8,416.93 per QALY. The majority of scenarios evaluated in the PSA were within the cost-effectiveness threshold (73%). In this subgroup, ADT alone was not considered to be cost-effective (Figure 2).

**Figure 3:**
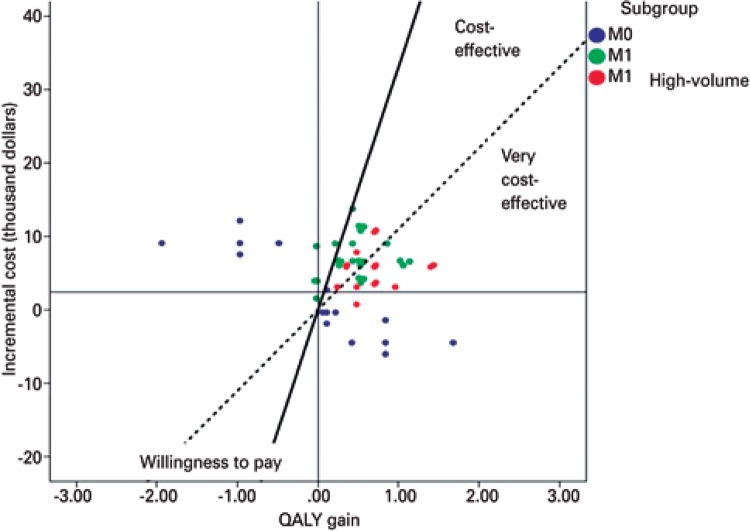
Cost-effectiveness of docetaxel plus androgen deprivation therapy for prostate cancer

M0: non-metastatic; M1: all metastatic; QALY quality-adjusted life-year.

Figure 3 presents all of the scenarios considered in the probabilistic sensitivity analysis including all of the patient subgroups evaluated in the model. This is a scatter plot in which each point represents a probabilistic analysis. The subgroups can be viewed together in different colors. The diagonal lines represent the cost-effectiveness thresholds according to the definitions of the World Health Organization:^(^ ^17^ ^)^ treatments that cost less than the value of one GDP per capita for each QALY gained are very cost-effective and treatments that cost up to three times the value of the GDP per capita for each QALY gained are cost-effective.

## DISCUSSION

The addition of chemotherapy to androgen deprivation therapy (ADT) represented a paradigm shift when initially used for the treatment of prostate cancer.^(^ ^6^ ^-^ ^8^ ^,^ ^11^ ^,^ ^18^ ^)^ However, there was a more pronounced practice change for patients with high-volume or more aggressive metastatic disease as a result of the differences between the GETUG-AFU 15 and CHAARTED studies.^(^ ^6^ ^,^ ^18^ ^)^ The first study included about 30% of patients with high-volume disease and did not find a benefit, while the second study included 70% of individuals with high-volume disease and found a statistically significant benefit.^(^ ^6^ ^,^ ^11^ ^)^

The aging population in Brazil may lead to an increase in the number of individuals with cancer.^(^ ^19^ ^)^ Furthermore, the development of potentially expensive new technologies may lead to a significant increase in cancer treatment costs.^(^ ^20^ ^)^ In Brazil, the expenses in cancer drug acquisition has increased almost three times in the past ten years.^(^ ^21^ ^)^ Approximately U$ 2.5 billion are spent each year on cancer drugs.^(^ ^21^ ^)^ Therefore, it is fundamental to evaluate the cost-effectiveness of the treatments.

Although the relevance of cost-effectiveness analyses are increasing, there is still great difficulty in defining an accepted ICER threshold.^(^ ^20^ ^)^ In an attempt to facilitate the interpretation of these data, the World Health Organization suggests that for a treatment to be cost-effective it must cost up to three times the value of GDP per capita per QALY gained, and for a treatment to be very cost-effective it must cost less than the GDP per capita.^(^ ^17^ ^)^ In the US the cost-effectiveness threshold is US$ 50,000 per QALY gained. This value is based on the costs required for hemodialysis treatment for patients with chronic renal failure. However, recent studies suggest this figure should be increased to US$ 100,000 or US$ 150,000.^(^ ^22^ ^)^ In the United Kingdom, the most commonly used threshold is £ 30,000.00 per QALY.

Considering these cost-effectiveness thresholds, we consider that the findings in this study are robust since they fall within the cost-effectiveness thresholds in the majority of the probabilistic sensitivity analysis performed. The most cost-effective treatment group was patients with high volume metastatic disease (up to six times more cost-effective compared to patients with non-metastatic neoplasms).

In the other hand, a Chinese study assessed the same question in China. The addition of docetaxel was not cost-effective for all patients with metastatic disease, although this treatment may be cost-effective for a minority of sensitivity analysis among patients with disease of high-volume. The incremental QALY found by the Chinese authors and by our group are quite similar.^(^ ^23^ ^)^ This endorses the replicability and robustness of our findings. Moreover, our study was the only that assessed the cost-effectiveness of the addition of docetaxel to ADT for non-metastatic disease.

Although the thresholds proposed by World Health Organization facilitate the interpretation of cost-effectiveness studies and the definition of the implementation of new technologies, these values consider only economic aspects of the population. Epidemiological, cultural, psychological, and spiritual aspects are not taken into account in this evaluation. For example, new treatment for a rare disease with limited therapeutic options may accept a higher cost-effectiveness threshold than a new treatment for a prevalent disease with good therapeutic options.

In addition, the discussion about the monetary value that should be invested in an individual’s life is very complex in Brazilian society. Furthermore, it is very difficult to standardize cost-effectiveness thresholds for individuals in different social strata.

There are fundamental questions regarding the interpretation of these findings that have not been fully addressed. One of the major limitations of this study was that the estimate of the quality of life based on data from the literature may be different from the Brazilian values and lead to possible changes in the findings. Since there are no Brazilian data to be used, it is extremely important to develop other quality of life studies in Brazil.

In our study, we included clinical data from randomized studies within populations outside of Brazil, which may differ from studies conducted in a Brazilian population. Therefore, the need for clinical studies in Brazil is required to confirm that data from international clinical studies are relevant.

## CONCLUSION

The addition of chemotherapy to the hormone treatment of prostate cancer is a cost-effective measure for patients with extensive (high-volume) metastatic disease. However, studies regarding the clinical effectiveness and quality of life are necessary to confirm these findings in Brazil.
